# CT Image Parameters for Predicting Surgical Risk and Outcome in Wilms Tumor

**DOI:** 10.7759/cureus.73613

**Published:** 2024-11-13

**Authors:** Supika Kritsaneepaiboon, Tanasap Rukkito, Pattama Tanaanantarak, Pornpun Sripornsawan, Surasak Sangkhathat, Najwa Yudhasompop, Rachaneekorn Cholsin, Polathep Vichitkunakorn

**Affiliations:** 1 Department of Radiology, Faculty of Medicine, Prince of Songkla University, Hat Yai, THA; 2 Department of Pediatrics, Faculty of Medicine, Prince of Songkla University, Hat Yai, THA; 3 Division of Pediatric Surgery, Department of Surgery, Faculty of Medicine, Prince of Songkla University, Hat Yai, THA; 4 Department of Pediatrics, Hatyai Hospital, Hat Yai, THA; 5 Department of Pediatrics, Maharaj Nakhon Si Thammarat Hospital, Nakhon Si Thammarat, THA; 6 Department of Family Medicine and Preventive Medicine, Faculty of Medicine, Prince of Songkla University, Hat Yai, THA

**Keywords:** image-based surgical risk factors, pediatric oncology, surgical complications, survival outcome, wilms tumor

## Abstract

Objective: This study determined preoperative image parameters for predicting surgical risk and outcome in Wilms tumor (WT).

Methods: A total of 55 patients with WT were enrolled and classified into surgically low-risk (SLR) and surgically high-risk (SHR) groups. The relationship between imaging findings and surgical risk factors was analyzed, and a survival analysis was performed.

Results: The number of patients in the SLR and SHR groups was 35 and 20, respectively. The abdominal aorta encasement, adrenal involvement, and tumor spillage of the computed tomography (CT) image parameters showed a statistically significant difference between the two groups (*p*-value = 0.021, 0.02, and < 0.01, respectively). Multivariable Cox regression analysis demonstrated that those three CT parameters significantly increased surgical risks (OR = 10.11 *p*-value = 0.043, OR = 7.61 *p*-value = 0.031, and OR = 55.57 *p*-value = < 0.001, respectively). The one-, two-, and five-year disease-free survival (DFS) rates were 83%, 78.2%, and 72.6%, respectively. The radiological parameters associated with poor survival were adrenal involvement and tumor spillage.

Conclusion: The abdominal aorta encasement, adrenal involvement, and tumor spillage in the preoperative CT image were strong evidence for predicting surgical risk and outcome in WT. These parameters could be beneficial for the surgeon in preoperative preparation and during the procedure.

## Introduction

Wilms tumor (WT) is the most common primary malignant renal tumor in children [1,2]. Currently, the following two mainstream algorithms are used in WT management: one from the International Society of Pediatric Oncology (SIOP), which promotes upfront chemotherapy before nephrectomy (SIOP WT 2001), aiming to reduce intraoperative spillage, and the other from the Children's Oncology Group (COG), which prefers primary nephrectomy unless the tumor is considered unresectable. In Thailand, the Thai Pediatric Oncology Group (TPOG) performed nationwide clinical trials and followed the National Wilms Tumor Study (NWTS), updated by the COG Renal Tumor Committee since 2001 [3]. However, there is still a lack of documented preoperative imaging criteria for predicting surgical risk and outcome.

Surgical staging or operative findings are required to determine and complete the NWTS protocols. According to the TPOG regimen for WT, most WTs are managed by primary nephrectomy followed by adjuvant chemotherapy unless the tumor is unresectable. Preoperative or upfront chemotherapy in WT cases with large tumor volumes is suggested after an image-guided biopsy of proven WT [4]. The presence of a large tumor or its rupture prior to surgery or during surgical manipulation can complicate the procedure. Therefore, preoperative imaging is essential for helping the surgeon identify these factors. Localized WT in Thailand typically exhibited a positive prognosis, especially when characterized by favorable tumor histology, with a five-year survival rate of more than 80% [5].

An image-based staging system was used for two solid pediatric tumors: hepatic tumors and neuroblastoma. For primary hepatic malignancy in children, the PRE-Treatment EXTent of tumor (PRETEXT) system was used to standardize imaging evaluation and risk stratification for children prior to the administration of neoadjuvant therapy. The clinical context and PRETEXT indicate poor prognosis [6]. For neuroblastoma, the international neuroblastoma risk group staging system (INRGSS) was based on imaging findings of the absence or presence of image-defined risk factors (IDRFs) [7,8]. IDRFs also predict the surgical risks of localized neuroblastoma and determine, during diagnosis, whether a patient should undergo surgery or chemotherapy.

However, these kinds of image-based surgical risk factors in WT are not well established. The reliability of image-based customized approaches may still be questionable, particularly given the limited diagnostic capacity. There is still no universally accepted protocol for imaging assessment in WT. This variability can lead to inconsistent data about surgical risk. It can lead to uncertainties in surgical planning and outcomes. Only one study has analyzed image-based surgical risk factors for WT and revealed that tumor size, displacement of the great vessels, and contralateral extension were significantly associated with surgical risks [9]. Nonetheless, this was not an in-depth study of the relationship between the tumor and neighboring vessels and organs; therefore, we created modified image checklists of WT to predict surgical risk and outcomes.

The main purpose of this study was to determine the preoperative imaging parameters (pre- or post-treatment images before surgery) to predict surgical risk and outcomes in patients with WT. The second purpose was to determine the imaging parameters (first-time diagnosis before any treatment) that predict disease survival in WT.

This article was previously presented as a scientific poster at the 58th Annual Meeting of the European Society of Pediatric Radiology on June 5-7, 2024.

## Materials and methods

Patients

This retrospective study was conducted by reviewing the medical records of patients (< 15 years of age) with histologically confirmed pediatric WT who underwent tumor resection at Songklanagarind Hospital, Hatyai Hospital, and Maharaj Nakhon Si Thammarat Hospital between April 1, 2005, and May 31, 2023. Access and use of these clinical data were approved by the Human Research Ethics Committee of the Faculty of Medicine, Prince of Songkla University (REC: 65-203-7-4). The data extracted from medical records in the computer-based hospital information system (HIS) and the Excel tool were utilized for data management. Patients with unavailable preoperative imaging findings, inadequate computed tomography (CT) scan quality, or insufficient diagnostic confidence were excluded.

The sample size calculation used the formula for the comparison of two proportions. The previous study derived the estimated proportion of high surgical risk in contralateral extension [9]. The calculated smallest sample size was 54 (power analysis = 80%, alpha value = 0.05, and beta value = 0.2). Data analysis included demographic data, radiological parameters, and surgical parameters. Demographic data included age at diagnosis, sex, body weight, body height, clinical presentation, histological subtype, staging, and distant metastasis. Radiological parameters included tumor size, midline crossing, vascular displacement, vascular encasement, involvement of the renal pedicle, ureter, adjacent organ structures, beyond renal capsule extension, enlarged nodes, and contralateral and tumor spillage. Surgical parameters included operative time, intraoperative bleeding, incomplete resection, intraoperative tumor spillage, major vessel injury, intra-abdominal organ injury, and postoperative complications. Data collection for clinical outcomes included one-, two-, and five-year disease-free survival (DFS) and one-, two-, and five-year overall survival (OS).

Imaging analysis

The three CT scanner models provided in our study and used during the study period were a 64-multislice Philips Brilliance CT scanner (Amsterdam, Netherlands), a 160-slice Canon Aquilion Prime CT scanner (Toshiba Medical, Otawara, Japan), and a 512-slice GE Revolution CT (GE Healthcare, Chicago, IL, USA). The CT parameters were calculated based on body weight, and the kilovoltage peaks (kVp) ranged between 80 and 120. The current in milliamperes (mA) was selected based on body weight using a dose-modulated technique in the Philips, Canon, and GE scanners. Intravenous non-ionic contrast medium was administered via an automated power injector in a peripheral vein. The contrast medium dose was 2 mL/kg, and the flow rate was adjusted between 1.5 and 4 mL/second depending on the needle gauge. The images acquired by the multi-slice CT scanners were reconstructed at section widths of 3 mm with 2-mm increments in children older than two years of age and 2 mm with 1-mm increments in children younger than two years of age.

**Figure 1:**
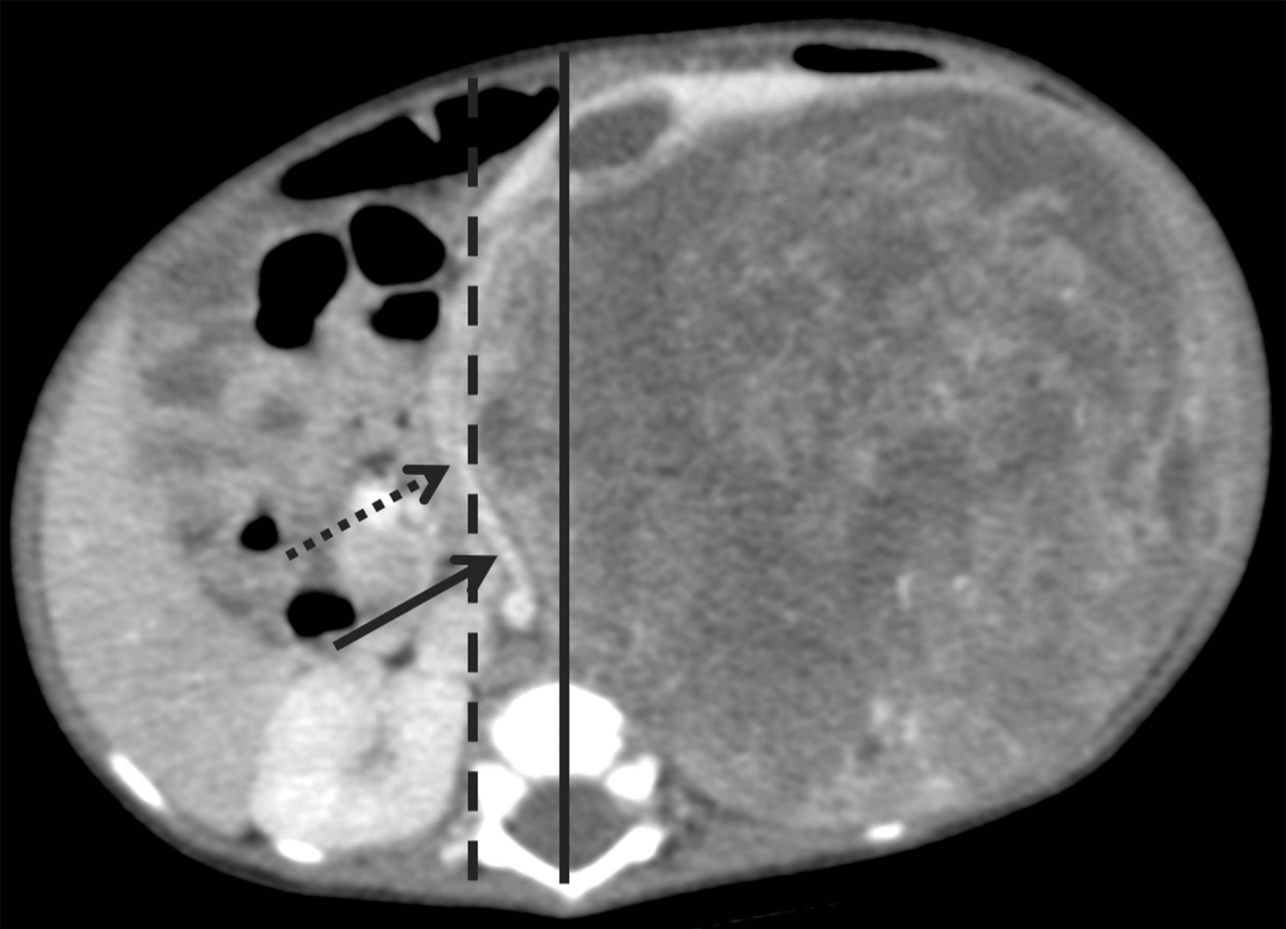
The axial CT image shows the definition of contralateral tumor extension (dashed line), left renal pedicle (dashed arrow), and left renal artery (straight arrow) involvement (midline of the vertebral body = straight line). CT: computed tomography

**Figure 2:**
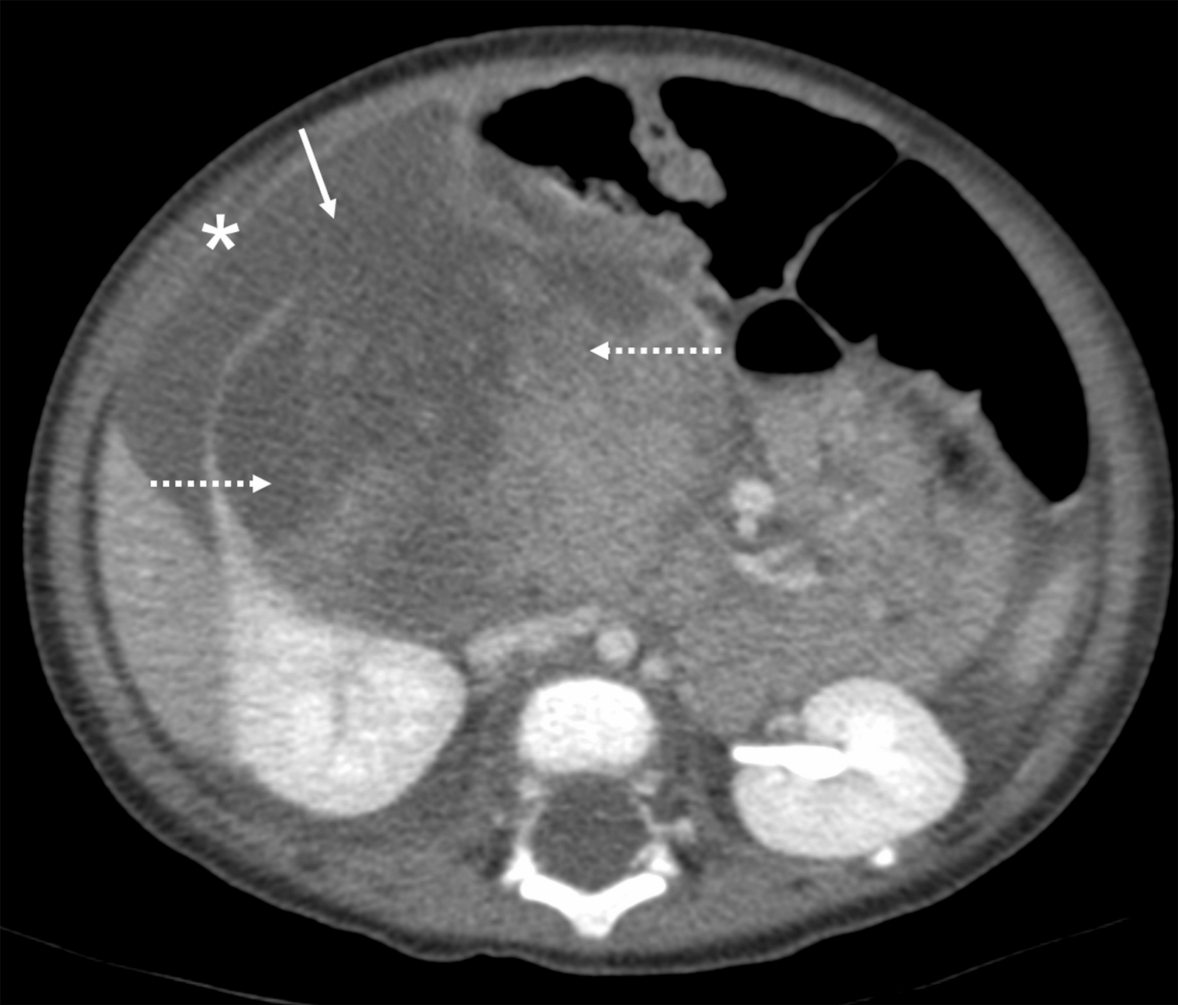
The axial CT shows WT in the right kidney (dashed arrows) with evidence of tumor spillage seen as discontinuous tumor edge (arrow) and ascites (*). CT: computed tomography; WT: Wilms tumor

**Figure 3:**
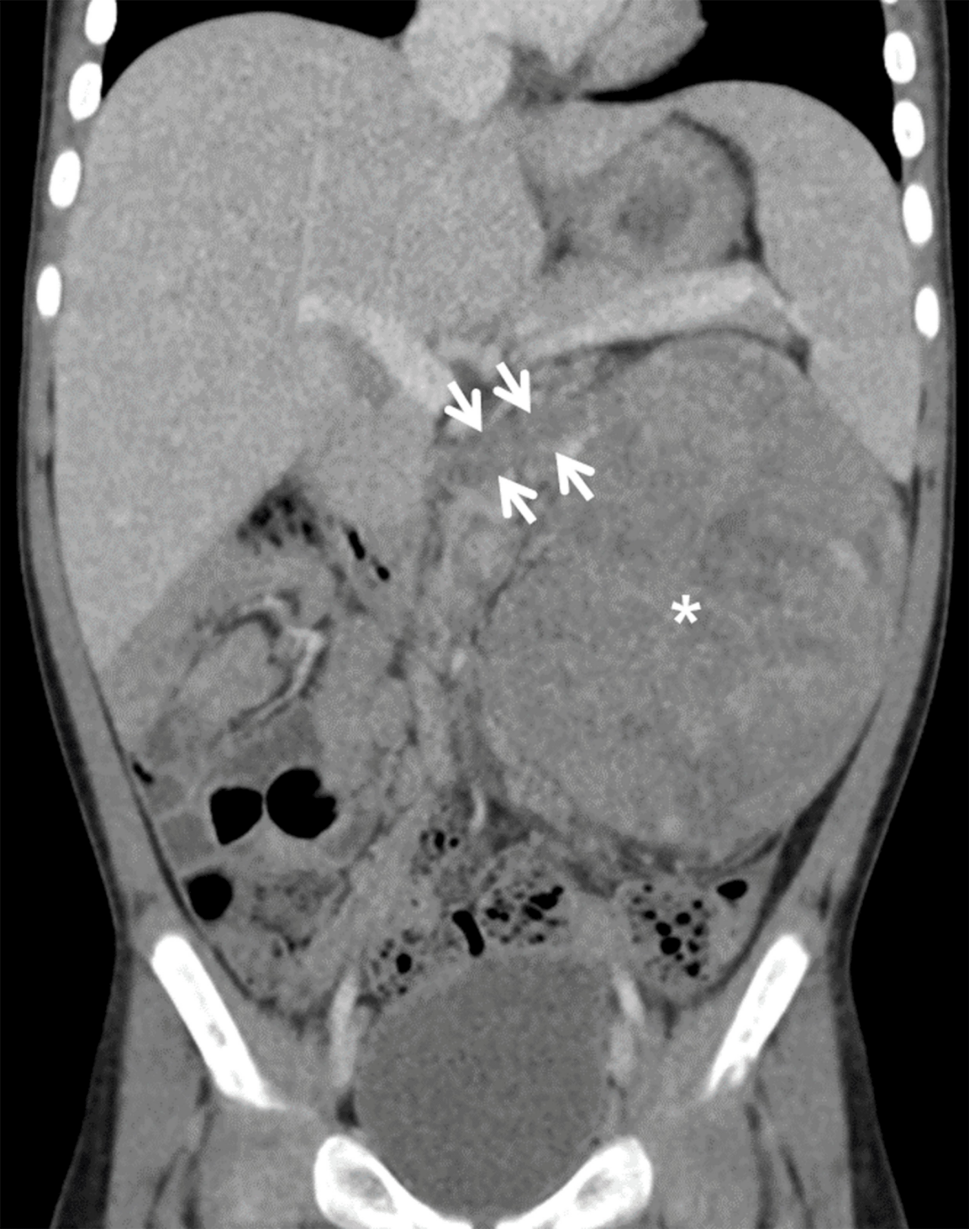
The coronal CT image shows WT in the left kidney (arrows) with a tumor thrombus extending to the left renal vein (asterisk). CT: computed tomography; WT: Wilms tumor

**Figure 4:**
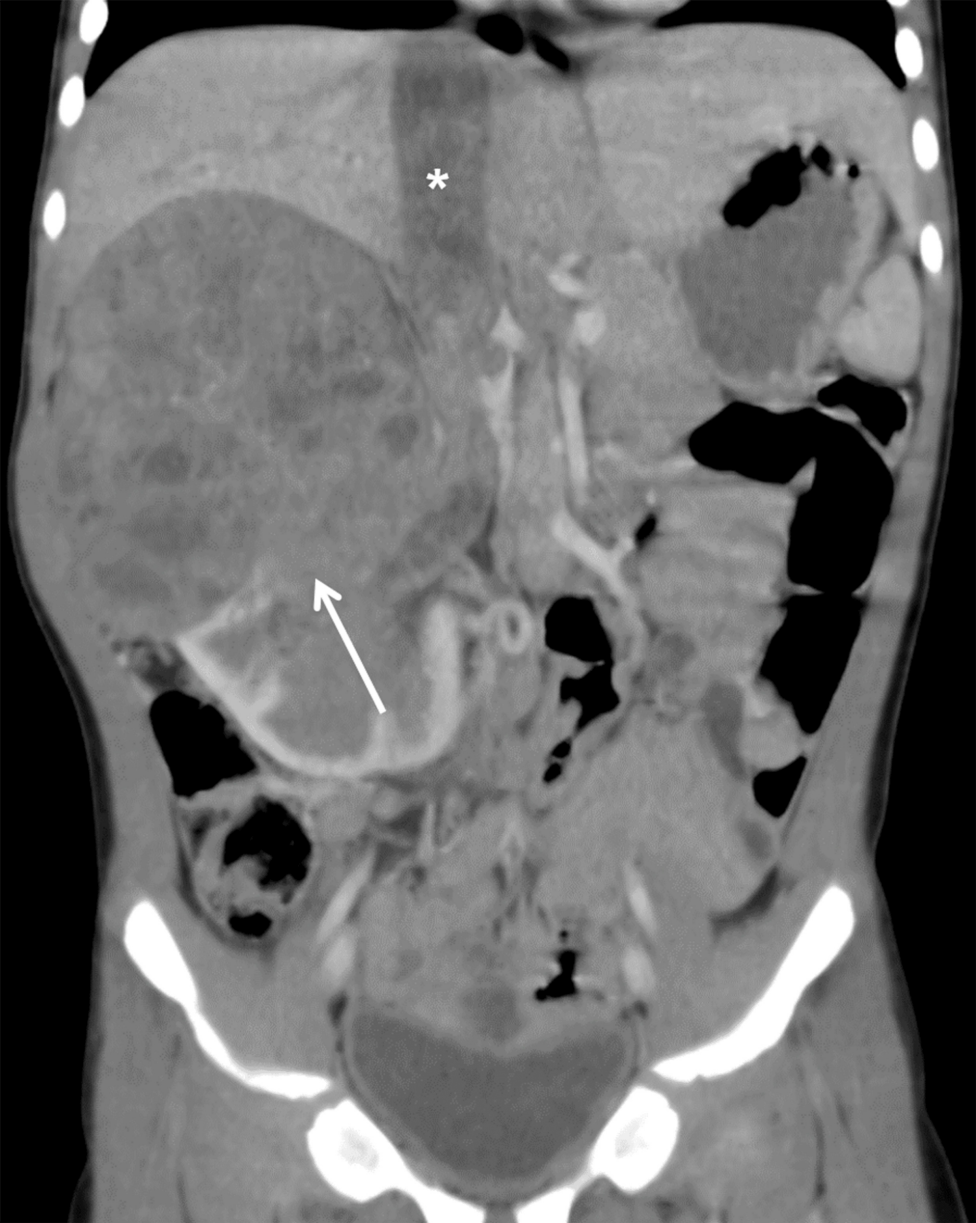
The coronal CT image shows WT in the right kidney (white arrow) and a tumor thrombus within the inferior vena cava (IVC) (*). CT: computed tomography; WT: Wilms tumor

**Figure 5:**
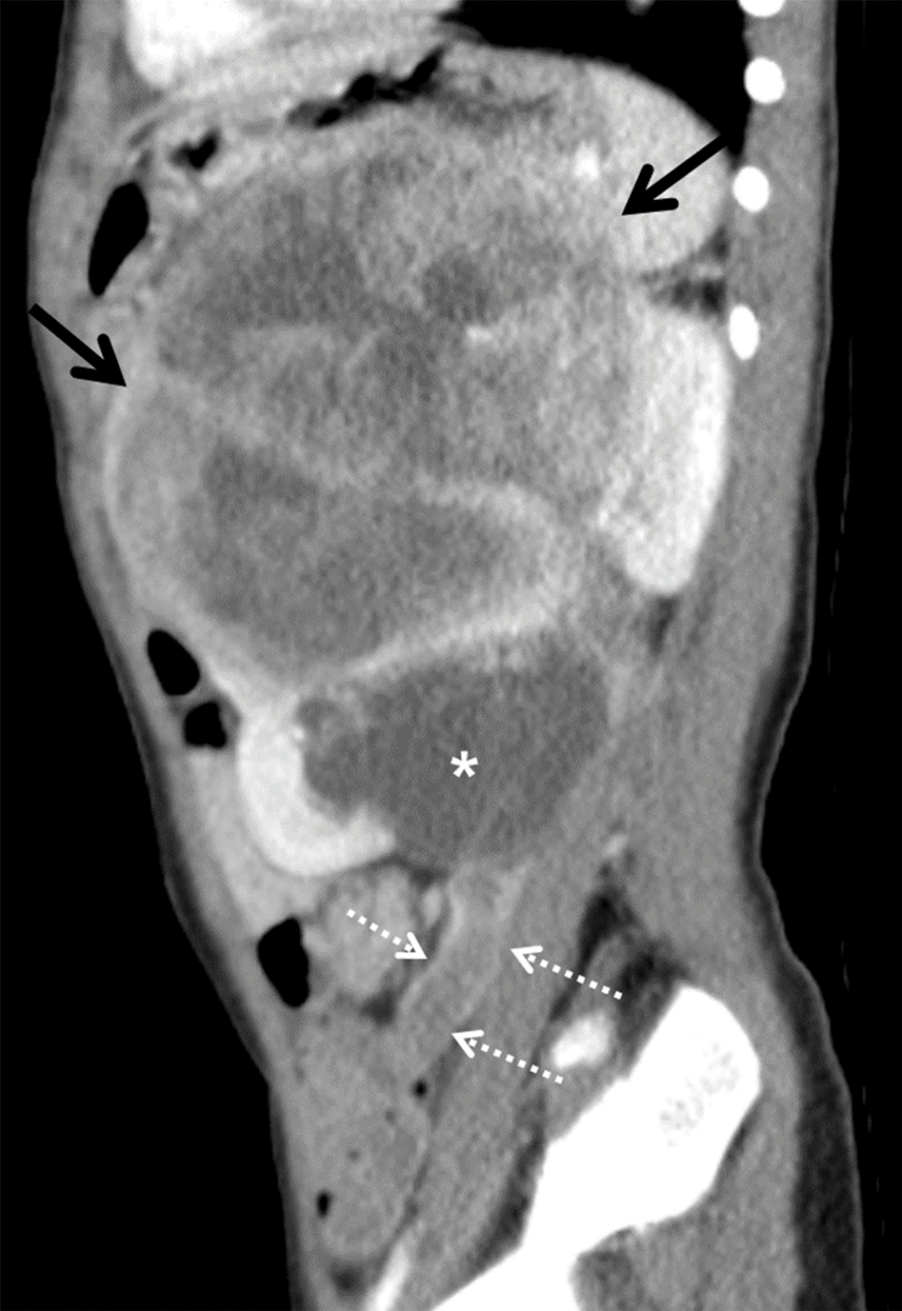
The sagittal CT image shows WT in the left kidney (arrows) with a tumor extension into the dilated left renal pelvis (asterisk) and the left ureter (dashed white arrows). CT: computed tomography; WT: Wilms tumor

Two pediatric radiologists with 17 and 14 years of experience independently reviewed the CT scans and reached a consensus. If there were any disagreement, the third reviewer would provide the consensus. The reviewers were blinded to the intraoperative findings. Table 1 and Figures 1-5 present the definitions of each CT parameter.

**Table 1:** Definition of each CT parameter CT: computed tomography; SMA: superior mesenteric artery

CT Parameter	Definition
Tumor size (absolute tumor size)	Measured in maximum size at three dimensions on the abdominal CT slice: in axial view, two dimensions were measured at horizontal diameter as the width (w) and at vertical diameter as the depth (d), and in coronal view, a dimension was measured at vertical diameter as the height (h)
Adjusted tumor size	By abdominal width (W), anteroposterior girth (D), and body height (H), respectively. Adjusted width = tumor width (w) / abdominal width Adjusted depth = tumor depth (d) / anteroposterior girth (D) Adjusted height = tumor height (h) / body height (H)
Tumor area	Tumor area = multiplication of two-dimensional tumor sizes (w × d) Ratio tumor area = tumor area/abdominal area (w × d) / (W × D)
Tumor volume	Tumor volume = multiplication of three-dimensional tumor sizes (w × d × h)
Adjusted tumor volume	w x d x h / body weight (BW)
Cross midline	Contralateral tumor extension was defined as a tumor extending beyond the midline of the vertebral body at the renal pedicle level (Figure 1)
Beyond renal capsule extension	Tumor outside the confines of the capsule (either by direct invasion of the perirenal soft tissue or extrarenal vasculature)
Tumor spillage	Poor delineated edge of the mass with peritumoral fat stranding and retroperitoneal fluid, ascites, or peritoneal implants Figure 2)
Involved renal pedicle	Encased renal pedicle > 180 degrees (Figure 1)
Vascular displacement	Displacement of great vessels was defined when a left-sided tumor compressed the abdominal aorta or a right-sided tumor compressed the inferior vena cava
Renal artery	> 180-degree encasement (Figure 1)
Renal vein	Thrombus, obliterated lumen > 50% (Figure 3)
Inferior vena cava	Thrombus, obliterated lumen > 50% (Figure 4)
Vascular encasement	Celiac trunk, SMA, or abdominal aorta (> 180-degree encasement)
Nodes	Regional, paraaortic, distant (> 1 cm in short axis diameter or any size with loss of oval shape)
Ureteral involvement	Periureteric fat stranding, wall thickening, or hydroureter (Figure 5)
Adjacent structures	Loss of normal fat plane separation with the adjacent organs or unable to identify adjacent structures. (adrenal gland, head of the pancreas, mesenteric root, 2nd to 3rd part duodenum)
Contralateral involvement	Bilateral Wilms tumor

We evaluated the CT findings performed within one month of the surgical date. For example, if the patient had a primary nephrectomy, we evaluated the pre-surgical CT, which is defined as a "pre-treatment CT." If the patient had an unresectable tumor and received upfront chemotherapy, we evaluated a post-chemotherapy CT or defined as "post-treatment CT." Unresectable tumors were defined based on imaging criteria or based on the surgeon's decisions. The suggestive imaging criteria for unresectable tumors were size > 10 cm, an inferior vena cava (IVC) thrombus, adherence to an adjacent organ, bilateral WTs, or extensive lung metastasis with compromised lung [4].

Relationship between surgical risks and image parameters

We determined the relationship between pretreatment and posttreatment CT images and surgical parameters categorized into surgically low- and high-risk groups. The surgically low-risk (SLR) group was defined as having a completely resected tumor, no intraoperative tumor spillage, no major vascular injury, or adjacent vital organ injury, whereas the surgically high-risk (SHR) group was defined as having an incompletely resected tumor, intraoperative tumor spillage, major vascular injury, or adjacent vital organ injury. The major vessel injury was defined as injury to the main renal vessels, abdominal aorta and their main branches, and IVC. The intraoperative hemorrhage was quantitatively assessed by the anesthesiologist and filled out the anesthesia record form. In the case of bilateral WT, we analyzed the ipsilateral tumor with nephrectomy.

Statistical analysis

Data analysis was performed using the R statistical program version 4.3.1 (The R Foundation, Vienna, Austria). Demographic data, such as continuous variables, were presented as mean (standard deviation) and median (interquartile range (IQR)), and categorical variables were presented as frequencies and percentages. For the normal distribution of the parameters, the result is displayed in the mean and the standard deviation. If the data is a non-normal distribution, it is customary to use the median and IQR instead of the mean and standard deviation. The association between categorical data and SLR and SHR groups was compared using the chi-square test or Fisher's exact test, and continuous data were compared using an independent t-test or Wilcoxon rank-sum test. The odds ratios (ORs) were used to present the strength of the association between CT imaging parameters and surgical risk groups. The statistical significance was considered when the *p*-value was < 0.05.

The log-rank test determined the association between each CT parameter and patient survival. Multivariable analysis of survival predictors was performed using Cox proportional hazards regression analysis. The hazard ratios (HRs) used to estimate the risk of poor survival outcomes are relatively close to the significant or high-risk imaging parameters. Statistical significance was set at *p*-value* *< 0.05.

## Results

Patient characteristics

A total of 58 patients were diagnosed with WT in this study. Three patients were excluded due to not having undergone surgery (one patient) and incomplete intraoperative information (two patients). Therefore, 55 patients were enrolled and divided into two groups: the SLR group with 35 patients and the SHR group with 20 patients.

Table 2 presents the patients' demographic data. Body height, clinical presentation, and tumor stage differed significantly between the SLR and SHR groups (*p*-value = 0.032, 0.010, and 0.006, respectively).

**Table 2:** Demographic data in the surgically high-risk and surgically low-risk groups * p-value is considered significant (p-value < 0.05). † All values in parentheses represent means, except for the demographic data on age, body weight, and height, which are presented as medians (IQR). IQR: interquartile range; kg: kilograms; cm: centimeters

Demographic Data	Surgically Low-Risk Group (N = 35)^†^	Surgically High-Risk Group (N = 20)^†^	Total (N = 55)^†^	p-value*
Sex				0.055
Female	12 (34.3)	13 (65)	25 (45.5)	-
Male	23 (65.7)	7 (35)	30 (54.5)	-
Age (month) median (IQR)	19.9 (11.8,37.6)	33 (17.6,42.9)	25.3 (13.3,40.4)	0.159
Body weight (kg) median (IQR)	10.8 (8.6,13.3)	12.2 (9.9,15.9)	11 (9.2,14.1)	0.151
Height (cm) median (IQR)	80 (67.8,90)	93 (81.2,103.8)	85.8 (73.1,97.9)	0.032*
Clinical presentation				0.010*
Palpable abdominal mass without related symptom	29 (82.9)	10 (50)	39 (70.9)	-
Palpable abdominal mass with related symptom	5 (14.3)	10 (50)	15 (27.3)	-
Nonpalpable abdominal mass	1 (2.9)	0 (0)	1 (1.8)	-
Histological subtype				0.999
Favorable	34 (97.1)	20 (100)	54 (98.2)	-
Unfavorable	1 (2.9)	0 (0)	1 (1.8)	-
Stage				0.006*
I-II	20 (57.1)	3 (15)	23 (41.8)	-
III-V	15 (42.9)	17 (85)	32 (58.2)	-
Lung metastasis				0.175
No	33 (94.3)	16 (80)	49 (89.1)	-
Yes	2 (5.7)	4 (20)	6 (10.9)	-
Upfront chemotherapy				0.739
No	18 (51.4)	12 (60)	30 (54.5)	-
Yes	17 (48.6)	8 (40)	25 (45.5)	-

The most common clinical symptom was a palpable abdominal mass without a related symptom, which was more prominent in the SLR group (29 cases, 82.9%), whereas a palpable abdominal mass with a related symptom was prominent in the SHR group (10 cases, 50%). Only one case presented with a non-palpable abdominal mass due to genitourinary screening in WT, aniridia, genitourinary anomalies, and range of developmental delays (WAGR) syndrome.

Sex, age at diagnosis, body weight, and histological subtype did not differ significantly between the SLR and SHR groups (*p*-value = 0.055, 0.159, 0.151, and 0.999, respectively).

In this study, 30 patients underwent primary nephrectomy (or no upfront chemotherapy) after initial CT (18 in the SLR group and 12 in the SHR group, and no statistically significant difference between the two groups) (Table 2). Of the 12 patients in the SHR group, nine underwent emergency nephrectomy due to acute abdominal pain, unstable hemodynamics, decreased hematocrit level, and evidence of ruptured WT on CT.

This study included six patients with stage IV WT, all of whom had lung metastases. Of these, two were in the SLR group and four in the SHR group, with no statistically significant difference between the two groups (Table 2). Almost all patients with stage III and IV WT received adjuvant radiotherapy after surgery, with two exceptions: one stage III patient who was lost to follow-up and one stage IV patient whose clinical condition was too unstable for radiotherapy.

Abdominal CT image parameters

Tables 3, 4 present abdominal CT image parameters analyzed using univariate and multivariate methods. Three imaging parameters showed statistically significant differences between the SLR and SHR groups, including abdominal aorta encasement, adrenal involvement, and tumor spillage (OR = 10.11, *p*-value = 0.043; OR = 7.61, *p*-value = 0.031; and OR = 55.57, *p*-value < 0.001, respectively).

**Table 3:** CT findings in the surgically high-risk and surgically low-risk groups * p-value is considered significant (p-value < 0.05). † Values in parentheses represent N (%). cm: centimeters; SD: standard deviation; IQR: interquartile range; cm^2^: square centimeter; mL: milliliter; kg: kilogram; SMA: superior mesenteric artery

CT Parameters	Surgically Low-Risk Group (N = 35)	Surgically High-Risk Group (N = 20)	p-value*
Tumor width (cm) mean (SD)	8.7 (2.4)	9.2 (2.6)	0.473
Tumor depth (cm) mean (SD)	9 (2.6)	9.7 (2.5)	0.335
Tumor height (cm) mean (SD)	10.5 (3)	11.8 (2.7)	0.129
Adjusted tumor width median (IQR)	0.5 (0.4,0.6)	0.5 (0.4,0.6)	0.896
Adjusted tumor depth median (IQR)	0.7 (0.6,0.8)	0.7 (0.6,0.8)	0.896
Adjusted tumor height mean (SD)	0.1 (0)	0.1 (0)	0.856
Pre-surgery maximum diameter (cm) mean (SD)	10.7 (3)	11.9 (2.7)	0.133
Maximum diameter (cm) mean (SD)			0.348
< 12	25 (71.4)	11 (55)	-
> 12	10 (28.6)	9 (45)	-
Absolute tumor size area (cm^2^) mean (SD)	84.5 (39.3)	95.1 (45.6)	0.370
Ratio tumor area median (IQR)	0.4 (0.3,0.4)	0.4 (0.3,0.5)	0.909
Tumor volume (mL) median (IQR)	943.5 (610.1,1158.6)	1103.7 (613.8,1528)	0.323
Adjusted tumor volume median (IQR) (mL/kg)	11.9 (7.6,14.5)	11 (7.6,17.4)	0.823
Cross midline^†^	24 (68.6)	13 (65)	0.999
Beyond renal capsule extension^†^	15 (42.9)	13 (65)	0.194
Tumor spillage^†^	1 (2.9)	12 (60)	< 0.001*
Renal pedicle involvement^†^	29 (82.9)	17 (85)	0.999
Vascular displacement^†^	24 (68.6)	14 (70)	0.999
Renal arterial involvement^†^	17 (48.6)	11 (55)	0.858
Renal vein involvement^†^	17 (48.6)	9 (45)	0.999
Inferior vena cava involvement^†^	6 (17.1)	5 (25)	0.503
Abdominal aorta encasement^†^	2 (5.7)	6 (30)	0.021*
Celiac or SMA encasement^†^	3 (8.6)	3 (15)	0.657
Regional nodal involvement^†^	22 (62.9)	12 (60)	0.999
Paraaortic nodal involvement^†^	7 (20)	2 (10)	0.462
Ureter involvement^†^	4 (11.8)	3 (15.8)	0.691
Adrenal involvement^†^	4 (11.4)	8 (40)	0.020*
Pancreas involvement^†^	10 (28.6)	9 (45)	0.348
Duodenal involvement^†^	5 (14.3)	4 (20)	0.709
Mesenteric root involvement^†^	1 (2.9)	1 (5)	0.99
Bilateral Wilms^†^	8 (22.9)	2 (10)	0.297

**Table 4:** Multivariable analysis of CT findings between the surgically low-risk and surgically high-risk groups * p-value is considered significant (p-value < 0.05). OR: odds ratio; CI: confidence interval

Variables	Subgroups	Adjust OR	95% CI		p-value*
			Lower	Upper	
Abdominal aorta involvement	No	Reference	-	-	0.043*
	Yes	10.11	1.08	114.12	
Adrenal involvement	No	Reference	-	-	0.031*
	Yes	7.61	1.21	55.02	
Tumor spillage	No	Reference	-	-	< 0.001*
	Yes	55.57	7.53	1222.59	

Intraoperative findings and surgical outcomes

Table 5 presents the intraoperative findings and surgical outcomes. The operative time and intraoperative bleeding showed statistically significant differences between the SLR and SHR groups (*p*-value = 0.002 and < 0.001, respectively). Of all the patients, only two were incompletely resected, with no statistically significant differences between the SLR and SHR groups (*p*-value = 0.128). The most common postoperative complication was gut obstruction, all of which were small bowel obstructions that failed conservative treatment and ended with surgical management, with no statistically significant difference between the SLR and SHR groups (*p*-value = 0.616).

**Table 5:** Surgical parameters in the surgically high-risk and surgically low-risk groups * p-value is considered significant (p-value < 0.05). Min: minute; SD: standard deviation; mL: milliliter; kg: kilogram; IQR: interquartile range

Surgical Parameters	Surgically Low-Risk Group (n = 35)	Surgically High-Risk Group (n = 20)	p-value*
Operative time (min) median (IQR)	227.5 (192,255)	280 (247.5,320)	0.002*
Intraoperative bleeding (mL/kg) median (IQR)	7.1 (3.1,12.3)	17.7 (11.1,35.1)	< 0.001*
Incomplete resection			0.128
No	35 (100)	18 (90)	-
Yes	0 (0)	2 (10)	-
Postoperative complication			
Gut obstruction			0.616
No	33 (94.3)	18 (90)	-
Small bowel	2 (5.7)	2 (10)	-

Survival analysis

The median follow-up period of DFS was 3.26 years (39.06 months, IQR 7.24-77.12). The one-, two-, and five-year DFS rates were 83.2%, 78.2%, and 72.6%, respectively. The median follow-up period of OS was 4.257 years (51.08 months, IQR 10.96-120.74 months). The one-, two-, and five-year OS rates were 93.9%, 91.4%, and 88.5%, respectively. During the follow-up period, five patients died, and 13 patients experienced recurrence or metastases. The mean time to recurrence or metastasis was 1.37 ± 1.614 years, with a median of 0.799 years (IQR 0.487-1.229 years). The radiological parameters associated with poor survival outcomes were adrenal involvement at the one- and five-year DFS and tumor spillage at the one-year OS (Table 6).

**Table 6:** One- and five-year disease-free survival (DFS) and one- and five-year overall survival (OS) according to CT and clinical parameters * p-value is considered significant (p-value < 0.05). † Unavailable first or pre-treatment CT image in one patient. The total number of patients for survival analysis for CT parameters was 54. DFS: disease-free survival; OS: overall survival; SMA: superior mesenteric artery

CT Parameters		DFS (n)	1-Y DFS (%)	Log-Rank p-value*	5-Y DFS (%)	Log-Rank p-value*	OS (n)	1-Y OS (%)	Log-Rank p-value*	5-Y OS (%)	Log-Rank p-value*
Overall	-	54^†^	83.2	-	72.6	-	54^†^	93.9	-	88.5	-
Maximum diameter	< 12 cm	35	82.9	0.9	66.29	0.4	35	96.88	0.3	88.42	0.8
	≥ 12 cm	19	83.5	-	83.51	-	19	88.82	-	88.82	-
Tumor volume	< 1000 mL	25	80.86	0.7	74.64	0.9	25	95.65	0.7	89.28	0.8
	≥ 1000 mL	29	85.01	-	71.41	-	29	92.5	-	88.32	-
Cross midline	No	15	77.0	0.5	67.4	0.6	15	91.67	0.8	82.5	0.6
	Yes	39	85.65	-	74.86	-	39	94.66	-	91.28	-
Beyond renal capsule	No	26	86.9	0.5	70.9	0.99	26	96.0	0.6	90.4	0.6
	Yes	28	79.77	-	74.79	-	28	91.92	-	87.08	-
Renal pedicle involvement	No	7	83.3	0.9	66.7	0.8	7	100	0.5	83.3	0.8
	Yes	47	83.2	-	73.9	-	47	92.97	-	90.06	-
Vascular displacement	No	12	90.91	0.5	79.5	0.5	12	100	0.3	88.9	0.8
	Yes	42	81.16	-	70.93	-	42	92.11	-	88.94	-
Renal arterial involvement	No	25	82.08	0.9	71.1	0.8	25	95.83	0.6	84.48	0.6
	Yes	29	84.64	-	74.23	-	29	92.35	-	92.35	-
Renal vein involvement	No	22	81.34	0.7	70.8	0.7	22	90.68	0.4	79.93	0.1
	Yes	32	84.55	-	73.89	-	32	96.77	-	96.77	-
IVC involvement	No	38	88.47	0.07	77.95	0.1	38	97.3	0.1	89.86	0.5
	Yes	16	68.9	-	57.4	-	16	84.4	-	84.4	-
Celiac or SMA encasement	No	49	83.35	0.8	71.4	0.8	49	95.32	0.1	89.21	0.4
	Yes	5	80.0	-	80.0	-	5	80.0	-	80.0	-
Abdominal aorta involvement	No	46	82.60	0.8	69.16	0.4	46	94.96	0.4	88.36	0.8
	Yes	8	87.5	-	87.5	-	8	87.5	-	87.5	-
Regional or paraaortic node	No	16	80.2	0.5	55.0	0.08	16	85.6	0.2	74.9	0.1
	Yes	38	84.89	-	80.85	-	38	97.3	-	93.69	-
Ureter involvement	No	46	82.3	0.9	69.77	0.6	46	95.03	0.3	88.61	0.6
	Yes	7	85.7	-	85.7	-	7	85.7	-	85.7	-
Adrenal involvement	No	41	88.85	0.04*	79.1	0.04*	41	97.44	0.07	90.7	0.3
	Yes	13	63.5	-	47.6	-	13	82.1	-	82.1	-
Pancreatic involvement	No	35	80.88	0.6	72.56	0.9	35	93.71	0.99	85.32	0.5
	Yes	19	87.97	-	73.1	-	19	94.4	-	94.4	-
Portal hepatis involvement	No	50	86.21	0.04*	N/A	N/A	50	95.43	0.07	89.5	0.3
	Yes	4	50.0	-	N/A	-	4	75.0	-	75.0	-
Duodenal involvement	No	46	85.08	0.4	76.05	0.2	46	95.1	0.3	88.79	0.7
	Yes	8	72.9	-	48.6	-	8	87.5	-	87.5	-
Bilateral Wilms tumor	No	43	81.77	0.7	75.71	0.6	43	95.23	0.5	92.26	0.2
	Yes	11	90.91	-	60.6	-	11	87.5	-	72.9	-
Tumor spillage	No	44	84.21	0.5	71.47	0.9	44	97.62	0.02	91.1	0.1
	Yes	10	80.0	-	80.0	-	10	77.1	-	77.1	-
Surgical risk	Low	35	83.61	0.8	71.83	0.9	35	96.97	0.2	89.00	0.7
	High	19^†^	82.59	-	75.1	-	19^†^	87.97	-	87.97	-
Intraoperative bleeding	< 10 mL/kg	24	86.36	0.5	80.97	0.3	24	95.65	0.6	90.34	0.6
	> 10 mL/kg	27	77.4	-	66.3	-	27	91.23	-	85.86	-
Lung metastasis	No	48	85.55	0.2	73.71	0.5	48	95.20	0.2	89.23	0.5
	Yes	6	66.70	-	66.70	-	6	83.31	-	83.31	-
Primary nephrectomy	No	25	80.81	0.6	63.53	0.3	25	96.50	0.4	84.32	0.5
	Yes	30	85.23	-	80.71	-	30	90.32	-	92.13	-

The one- and five-year DFS rates in patients with primary nephrectomy were 85.2% and 80.7%, respectively, which were not statistically significantly different from those without primary nephrectomy (Table 6). The one- and five-year OS rates in patients with primary nephrectomy were 96.5% and 92.1%, respectively, which were not statistically significantly different from those without primary nephrectomy (Table 6).

While DFS rates were lower in patients with lung metastasis (66.7% for both one- and five-year DFS) compared to those without lung metastasis (85.7% for one-year DFS and 73.7% for five-year DFS), these differences were not statistically significant. Similarly, OS rates were lower in patients with lung metastasis (83.3% for both one- and five-year OS) than in those without lung metastasis (95.2% for one-year OS and 89.2% for five-year OS), but these differences were also not statistically significant (Table 6).

Stepwise multivariable analysis revealed that only preoperative tumor spillage was independently associated with poor one-year OS (adjusted HR = 12.7, 95% CI = 0.81-198.81, *p*-value = 0.07). Conversely, the IVC and adrenal involvement were CT findings associated with poor one-year OS without statistical significance (adjusted HR = 2.38, 95% CI = 0.11-53.32, *p*-value = 0.585 and adjusted HR = 4.9, 95% CI = 0.21-113.87, *p*-value = 0.322, respectively).

## Discussion

We found a higher surgical risk among females than males; however, there were no statistically significant differences in sex between the SLR and SHR groups (*p*-value = 0.055). This was different from the study conducted by Barber et al., who reported that the ratios between females and males in patients with ruptured tumors and those without ruptured tumors were approximately 3:3 and 16:19, respectively, indicating that males and females were at equal surgical risk [10].

The age at diagnosis in the SLR and SHR groups was approximately 19.9 and 33 months, respectively, with no statistically significant differences (*p*-value = 0.159). The ages of patients without ruptured tumors and with ruptured tumors in Barber et al.'s study were approximately 3.6 and 3.8 years, respectively, and the age of ruptured tumors was slightly older than in our study [10]. Furthermore, older age at diagnosis, particularly >4 years, was independently associated with worse OS [11]. Besides that, the patient's height was significantly higher in the SHR group. It could be due to the fact that the tumor size is relatively larger in older children (Table 2); the risk of bleeding and tumor rupture may become higher.

No previous study data showed the clinical symptom presentations in the SLR and SHR groups. We analyzed the clinical presentations and categorized them into three groups: patients presenting with a palpable abdominal mass without a related symptom, patients presenting with a palpable abdominal mass with a related symptom, and patients with a non-palpable abdominal mass. The presence of more abdominal mass-related symptoms indicated a higher surgical risk. Related symptoms include gross hematuria, high blood pressure, or acute abdominal pain.

The tumor stage was also significantly associated with surgical risk (*p*-value = 0.006). Stages I-II were more pronounced in the SLR group, whereas stages III-V were more pronounced in the SHR group. However, only one-third of advanced stages present with tumor recurrence [11].

The relationship between tumor size and surgical difficulty or tumor spillage has been previously discussed. In an NWTS study, Ritchey et al. reported that a tumor diameter of >10 cm was associated with an increased risk of surgical complications [12]. Gow et al. reported that a tumor size of >12 cm was a risk factor for intraoperative tumor spillage [13]. Barber et al. reported that a tumor volume of >1000 mL was significantly associated with intraoperative tumor spillage [10]. Fukuzawa et al. showed that a ratio of tumor area to abdominal area (T/A ratio) of >0.5 might be a predictive factor for intraoperative tumor rupture in unilateral WT with favorable histology [14]. However, we found that all types of tumor size and volume were not significantly different between the SLR and SHR groups. This may be because the tumors in our study were initially large (994 mL in the SLR group and 1104 mL in the SHR group; *p*-value = 0.323) and did not respond well to chemotherapy. The mean tumor volume decreased by only 25.3 mL after upfront chemotherapy. Furthermore, previous studies showed that a larger tumor size (>15 cm, >11.5 cm, and >270 mL in Ekuk et al., Li et al., and Joseph et al.'s studies, respectively) was a poor prognostic factor or associated with worse OS, while either tumor size or volume was associated with worse OS in our study [15-17].

Vascular and adjacent structural involvement also affect surgical risk and outcomes. Our study found that when the WT had abdominal aorta encasement or adrenal gland involvement, the risk of surgical outcomes increased (OR = 10.11, *p*-value = 0.043 and OR = 7.61, *p*-value = 0.031, respectively), which had not been previously reported. Only one study by Oue et al. reported that displacement of the great vessels was significantly higher in the SHR group than in the SLR group (*p*-value = 0.0176) [9]. Adrenal involvement in our study was associated with poor survival, whereas atrioventricular thrombus and IVC occlusion represented poor event-free survival and OS in the study by Qureshi et al. [18]. The surgical guidelines in our country also recommend combined adrenal resection when the tumor is in contact with the adrenal gland or found adrenal infiltration in the surgical field.

Our study showed that tumor spillage on CT was significantly different (OR = 55.57, *p*-value < 0.001) between the SLR and SHR groups. Moreover, of the 20 patients in the SHR group, preoperative and intraoperative spillage was observed in 11 and 10 patients, respectively. Tumor spillage or preoperative tumor rupture was also associated with an increased risk of postoperative local recurrence or metastasis, but neither was associated with poor OS [19,20].

Regarding the operative time, our study found statistically significant differences between the SLR and SHR groups (227.5 vs. 280 minutes, *p*-value = 0.02). This was similar to the findings of Oue et al., who reported that the operative time in the SLR group was lower than that in the SHR group (241 and 357 minutes, *p*-value = 0.0189) [9]. Intraoperative bleeding in the SLR and SHR groups in our study showed statistically significant differences (7.1 vs. 17.7 mL/kg, *p*-value < 0.001). Compared with Oue et al., they reported intraoperative bleeding of approximately 20.3 and 36.8 mL/kg between these two groups but no statistically significant differences (*p*-value = 0.389) [9]. However, they found that tumor size was significantly correlated with intraoperative bleeding.

When preoperative imaging shows abdominal aorta encasement, adrenal gland involvement, or tumor spillage, these CT parameters significantly influence surgical planning and patient outcomes. They also help surgeons prepare more effectively for the operation, reducing intraoperative bleeding, minimizing prolonged operative time, and decreasing postoperative complications. The suggested future research will include additional radiological parameters not covered by this study, such as lung metastasis, utilize computer aids to predict high-risk imaging parameters, analyze imaging predictors as part of the risk stratification for WT, and investigate the impact of preoperative chemotherapy affecting the CT parameters.

Limitations

Our study had a few limitations. First, it was retrospective in nature, with a few incomplete operative notes. Second, we had to recruit patients from other medical institutions because we did not have enough patients with WT at our institution, which did not meet the sample size requirements. Third, multivariable analysis could not be performed for some CT image parameters due to the small sample size of the positive findings. We recommend conducting a prospective study and recruiting more patients in regions other than Southern Thailand.

## Conclusions

Three preoperative abdominal CT images consisting of abdominal aorta encasement, adrenal involvement, and tumor spillage provided strong evidence for predicting surgical risk and outcomes in patients with WT. These CT parameters could be beneficial for the surgeon in preoperative preparation and during the procedure to minimize tumor spillage and beware of adjacent organ injury and complications. The operative time and intraoperative bleeding significantly differed between the SLR and SHR groups. Only preoperative tumor spillage on CT was significantly associated with a poor one-year OS. Future studies should analyze imaging predictors as risk stratifications in WT or use computer aids to predict imaging high-risk factors.
